# Diabetes and Technology for Increased Activity Study: The Effects of Exercise and Technology on Heart Rate Variability and Metabolic Syndrome Risk Factors

**DOI:** 10.3389/fendo.2013.00121

**Published:** 2013-09-19

**Authors:** Melanie I. Stuckey, Antti M. Kiviniemi, Robert J. Petrella

**Affiliations:** ^1^School of Kinesiology, The University of Western Ontario, London, ON, Canada; ^2^Aging, Rehabilitation and Geriatric Care Research Centre, Lawson Health Research Institute, London, ON, Canada; ^3^Department of Exercise and Medical Physiology, Verve Research, Oulu, Finland; ^4^Schulich School of Medicine and Dentistry, The University of Western Ontario, London, ON, Canada

**Keywords:** autonomic nervous system, exercise training, metabolic syndrome, mobile health, heart rate variability

## Abstract

This study tested the hypothesis that an 8-week exercise intervention supported by mobile health (mHealth) technology would improve metabolic syndrome (MetS) risk factors and heart rate variability (HRV) in a population with MetS risk factors. Participants (*n* = 12; three male; aged 56.9 ± 7.0 years) reported to the laboratory for assessment of MetS risk factors and fitness (VO_2max_) at baseline (*V*_0_) and after 8-weeks (*V*_2_) of intervention. Participants received an individualized exercise prescription and a mHealth technology kit for remote monitoring of blood pressure (BP), blood glucose, physical activity, and body weight via smartphone. Participants underwent 24-h ambulatory monitoring of R–R intervals following *V*_0_ and *V*_2_. Low and high frequency powers of HRV were assessed from the recording and the ratio of low-to-high frequency powers and low and high frequency powers in normalized units were calculated. One-way repeated measures analysis of variance showed that waist circumference (*V*_0_: 113.1 ± 11.0 cm, *V*_2_: 108.1 ± 14.7 cm; *p* = 0.004) and diastolic BP (*V*_0_: 81 ± 6 mmHg, *V*_2_: 76 ± 11 mmHg; *p* = 0.04) were reduced and VO_2max_ increased (*V*_0_: 31.3 ml/kg/min, *V*_2_: 34.8 ml/kg/min; *p* = 0.02) with no changes in other MetS risk factors. Low and high frequency powers in normalized units were reduced (*V*_0_: 75.5 ± 12.0, *V*_2_: 72.0 ± 12.1; *p* = 0.03) and increased (*V*_0_: 24.5 ± 12.0, *V*_2_: 28.0 ± 12.1; *p* = 0.03), respectively, with no other changes in HRV. Over the intervention period, changes in systolic BP were correlated negatively with the changes in R–R interval (*r* = −0.600; *p* = 0.04) and positively with the changes in heart rate (*r* = 0.611; *p* = 0.03), with no other associations between MetS risk factors and HRV parameters. Thus, this 8-week mHealth supported exercise intervention improved MetS risk factors and HRV parameters, but only changes in systolic BP were associated with improved autonomic function.

## Introduction

Cardiovascular diseases are the leading cause of death world-wide accounting for 48% of mortality from non-communicable diseases (1). Type 2 diabetes mellitus is an independent risk factor for cardiovascular diseases and cardiovascular complications are common in this patient population. Metabolic syndrome (MetS) is a clustering of risk factors including abdominal obesity, hypertension, dysglycemia, and dyslipidemia, which doubles the 5-year risk of developing cardiovascular diseases and increases the life-time risk of developing type 2 diabetes mellitus fivefold (2). Lifestyle interventions are currently recommended as first-line therapy for MetS (2, 3).

Heart rate variability (HRV) is a simple, non-invasive measure that can be used to quantify autonomic nervous system modulation (4). Diminished HRV predicts all cause and cardiovascular mortality (5–8) and impaired autonomic function may be especially dangerous for those with an already increased risk of developing cardiovascular disease. Patients with type 2 diabetes mellitus with low HRV have double the risk of mortality compared to those with normal HRV (5). Recent reviews have suggested that autonomic dysfunction may be involved in the development of MetS and further dysfunction may be associated with progression along the continuum of cardiovascular disease (9, 10). Cross-sectional analyses have shown that 24 h HRV is reduced in MetS populations (11, 12), which may further increase cardiovascular risk. There is evidence that lifestyle changes may have positive autonomic effects (13, 14) but, it is unknown whether changes in MetS risk factors with lifestyle modifications are associated with concomitant changes in HRV.

We recently completed a pilot study examining the effects of a prescriptive exercise intervention supported by mobile health (mHealth) technology to improve MetS risk factors and clinical markers of cardiovascular disease (15, 16). This paper reports on a subset of participants with sufficient data for assessment of HRV in a population presenting with MetS risk factors. Thus, the purpose was to test the hypothesis that an 8-week lifestyle intervention supported by mHealth technology would improve HRV and that changes in MetS risk factors would be related to changes in HRV parameters.

## Materials and Methods

Twenty-five participants volunteered and provided informed consent to participate in this study. Participants were included if they had at least two MetS risk factors according to ATPIII guidelines: waist circumference ≥88 cm (women) or 102 cm (men); resting systolic blood pressure (SBP) ≥135 mmHg and/or diastolic blood pressure (DBP) ≥85 mmHg; fasting plasma glucose ≥6.1 mmol/L; triglycerides ≥1.7 mmol/L; and high density lipoprotein cholesterol (HDL) ≤1.03 mmol/L (men) or 1.29 mmol/L (women) [National Cholesterol Education Program (NCEP) Expert Panel on Detection, Evaluation, and Treatment of High Blood Cholesterol in Adults (Adult Treatment Panel III) (17)]. Exclusion criteria were SBP > 180 mmHg and/or DBP > 110 mmHg; type 1 diabetes; history of myocardial infarction, angioplasty, coronary artery bypass, or cerebrovascular ischemia/stroke; symptomatic congestive heart failure; atrial flutter; unstable angina; unstable pulmonary disease; use of medications known to affect heart rate (HR) (such as beta blockers); second or third degree heart block; history of alcoholism, drug abuse, or other emotional cognitive or psychiatric problems; pacemaker; unstable metabolic disease; and orthopedic or rheumatologic problems that could impair the ability to exercise. One participant withdrew from the study shortly following baseline testing due to hospitalization for a respiratory illness unrelated to the study. Twenty-four participants (aged 56.6 ± 9.0 year; six male) reported to Gateway Rural Health Research Centre at baseline (*V*_0_) and after four (*V*_1_) and eight (*V*_2_) weeks of intervention. This study was approved by Institutional Review Board Services (Aurora, ON, Canada; #RP-2008).

At each visit, blood pressure (BP) was measured following a 5-min rest in the seated position with an automated BP cuff (BPTru™ VSM MedTech Ltd., Coquitlam, BC, Canada). Clinic BP was calculated as the average of the last two of three measures taken at 2-min intervals. Waist circumference was measured as the midpoint between the lower rib and iliac crest (cm) (18). Blood was drawn from the antecubital vein and samples were sent to a central processing lab (Gamma Dynacare, London, ON, Canada) for analysis of fasting plasma glucose, triglycerides, and HDL.

The Step Test Exercise Prescription (STEP™) was administered to estimate fitness (VO_2max_; ml/kg/min) and counsel participants regarding physical activity. The full protocol is reported elsewhere (19). Briefly, participants were instructed to step up and down a set of 2 steps 20 times at a comfortable pace. HR was measured immediately following the test by palpation of the radial artery and input into the equation for calculation of predicted VO_2max_. Exercise prescription followed American College of Sports Medicine guidelines (20), with a target exercise HR of 70–85% of age predicted maximum HR, depending on VO_2max_. Goals included increasing pedometer-monitored steps per day with the overall goal of achieving 10,000 steps per day (21). The exercise prescription and goals were updated at *V*_1_.

### Mobile health protocol

Details of mHealth biometric and activity monitoring and database security are reported elsewhere (15). Briefly, participants received a smartphone (Blackberry^®^ Curve 8300, Research in Motion, Waterloo, ON, Canada) equipped with health monitoring software (Healthanywhere™, IgeaCare Inc., Markham, ON, Canada), a Bluetooth™ enabled BP monitor (A&D Medical, UA-767PBT, San Jose, CA, USA), a glucometer (LifeScan OneTouch Ultra2™, Milpitas, CA, USA, with wireless Bluetooth™ adapter Polymap, PWR-08-03, Tucson, AZ, USA), and a pedometer (Omron, HJ-150, Kyoto, Japan). Blood glucose measures were to be submitted twice daily (fasted upon waking and non-fasted before bed), BP measures were to be submitted three times per week upon waking, pedometer steps were to be input nightly, and body weight input weekly. Real-time measurements were sent to a secure central database that was monitored regularly by researchers. Readings that were outside of pre-set limits triggered alarms that automatically sent a message to the study physician’s smartphone to follow up with the participant.

The participants underwent ambulatory 24-h recording of R–R intervals on their least active day during the week immediately following *V*_0_ and *V*_2_ visits. R–R interval recording was conducted using a HR monitor with sampling accuracy of 1 ms (Suunto Memory Belt, Vantaa, Finland). Data were downloaded from the monitor to a personal computer for subsequent analysis (Heart Signal Co, Oulu, Finland). Frequency domain analysis for 24-h HRV was calculated as the average of 1 h epochs. An autoregressive model (order 20) was used to estimate power spectrum density for low (0.04–0.15 Hz) and high frequency powers (0.15–0.4 Hz) (4). Low and high frequency powers were also expressed in normalized units by dividing the low and high frequency power, respectively, by the sum of the low and high frequency power and multiplying by 100. Recordings with <80% qualifying beats were excluded from analysis.

### Statistical analysis

SigmaPlot (Version 11.0, Systat Software, San Jose, CA, USA) was used for analysis. One-way repeated measures analysis of variance was used to examine changes in MetS risk factors and HRV parameters from *V*_0_ to *V*_2_. Normality was tested with the Shapiro–Wilks test. Low and high frequency powers of HRV were logarithmically transformed to achieve normal distribution. Spearman rank order correlation was used to examine correlation between change in MetS risk factors and change in HRV over the course of the intervention. Spearman rank order correlation was used since mean change was not normally distributed and since the sample size was small. Data are shown as mean ± SD and significance was set at *p* < 0.05.

## Results

Participant characteristics, clinic, and home-monitoring results for the full population have been reported elsewhere (15, 16). Twelve participants (aged 44–69 year) had acceptable 24 h HRV recordings at both *V*_0_ and *V*_2_. Baseline characteristics for this subgroup and changes in MetS risk factors are reported in Table 1. Similar to the full population, this subgroup showed reductions in waist circumference (*V*_0_: 113.1 ± 11.0 cm, *V*_2_: 108.1 ± 14.7 cm; *p* = 0.004) and DBP (*V*_0_: 81 ± 6 mmHg, *V*_2_: 76 ± 11 mmHg; *p* = 0.04) with no changes in SBP, fasting plasma glucose, triglycerides, or HDL. Additionally, VO_2max_ increased from 31.3 ml/kg/min at *V*_0_ to 34.8 ml/kg/min at *V*_2_ (*p* = 0.02). The average of pedometer steps per day entered during week 1 and 8 were calculated to determine physical activity at *V*_0_ and *V*_2_, respectively. Over the intervention period, daily steps increased from 6472 ± 1997 steps per day at *V*_0_ to 8376 ± 2285 steps per day at *V*_2_.

**Table 1:** **Participant characteristics and changes in metabolic syndrome risk factors from *V*_0_ to *V*_2_**.

	*V*_0_	*V*_2_	*p*-Value
*N*	12 (3 Male; 25%)		
Age (year)	56.9 ± 7.0		
WC (cm)	113.1 ± 11.0	108.1 ± 14.7	0.002*
SBP (mmHg)	139 ± 7	134 ± 12	0.15
DBP (mmHg)	81 ± 6	76 ± 11	0.04*
FPG (mmol/L)	5.5 ± 0.9	5.4 ± 1.0	0.59
TG (mmol/L)	1.85 ± 1.64	1.42 ± 0.78	0.22
HDL (mmol/L)	1.35 ± 0.35	1.41 ± 0.46	0.19
VO_2max_ (ml/kg/min)	31.3 ± 6.7	34.8 ± 8.7	0.02*

DBP, diastolic blood pressure; FPG, fasting plasma glucose; HDL, high density lipoprotein cholesterol; SBP, systolic blood pressure; TG, triglycerides; VO_2max_, predicted maximal oxygen capacity; WC, waist circumference, *p < 0.05.

High frequency power in normalized units increased from 24.5 ± 12.0 at *V*_0_ to 28.0 ± 12.1 at *V*_2_ (*p* = 0.03) and low frequency power in normalized units decreased from 75.5 ± 12.0 at *V*_0_ to 72.0 ± 12.1 at *V*_2_ (*p* = 0.03). There were no changes in other 24 h HRV variables (Table 2).

**Table 2:** **Twenty-hour heart rate variability before and after the 8-week intervention**.

	*V*_0_	*V*_2_	*p*-Value
RRI (ms)	822 ± 127	849 ± 131	0.29
HR (bpm)	75 ± 11	72 ± 11	0.08
lnLF (ms^2^)	6.39 ± 0.78	6.41 ± 0.85	0.73
lnHF (ms^2^)	5.18 ± 1.00	5.39 ± 1.09	0.11
LFnu	75.5 ± 12.0	72.0 ± 12.1	0.03*
HFnu	24.5 ± 12.0	28.0 ± 12.1	0.03*
LF/HF	3.9 ± 2.0	3.4 ± 2.6	0.22

bpm, Beats per minute; HF, high frequency power; HFnu, high frequency power in normalized units; HR, heart rate; LF, low frequency power; LFnu, low frequency power in normalized units; RRI, R–R interval, *significant with *p* < 0.05.

Correlation analysis showed that the change in SBP was inversely associated with the change in R–R interval (−0.600, *p* = 0.04) and positively associated with the change in HR (0.611, *p* = 0.03). There were no other relationships between changes in MetS risk factors and changes in HRV parameters (Table 3).

**Table 3:** **Correlation coefficients for changes in heart rate variability parameters and metabolic syndrome risk factors over the intervention period**.

	ΔRRI	ΔHR	ΔlnLF	ΔlnHF	ΔLFnu	ΔHFnu	ΔLF/HF
Age	0.242	−0.246	−0.112	−0.203	0.263	−0.263	0.305
	0.43	0.43	0.71	0.51	0.39	0.39	0.32
ΔWC	−0.152	0.196	0.349	0.543	−0.536	0.536	−0.455
	0.62	0.53	0.25	0.06	0.07	0.07	0.13
ΔSBP	−0.600	0.611	−0.294	−0.046	−0.221	0.221	−0.329
	0.04*	0.03*	0.34	0.87	0.47	0.47	0.28
ΔDBP	−0.305	0.378	−0.273	−0.119	−0.070	0.070	−0.105
	0.32	0.21	0.38	0.70	0.82	0.82	0.73
ΔFPG	−0.061	0.066	−0.424	0.084	−0.529	0.529	−0.518
	0.83	0.83	0.16	0.78	0.07	0.07	0.08
ΔTG	−0.326	0.372	−0.462	−0.329	−0.151	0.151	−0.214
	0.28	0.22	0.12	0.28	0.62	0.62	0.48
ΔHDL	−0.063	−0.050	−0.102	−0.287	0.182	−0.182	0.147
	0.83	0.87	0.73	0.35	0.56	0.56	0.64
ΔVO_2max_	0.123	−0.191	0.203	0.168	0.007	−0.007	−0.007
	0.68	0.54	0.51	0.59	0.97	0.97	0.97
ΔSteps	0.476	−0.438	0.035	0.406	−0.336	0.336	−0.280
	0.11	0.14	0.90	0.18	0.27	0.27	0.36

DBP, diastolic blood pressure; FPG, fasting plasma glucose; HDL, high density lipoprotein cholesterol; HF, high frequency power; HFnu, high frequency power in normalized units; HR, heart rate; LF, low frequency power; LFnu, low frequency power in normalized units; RRI, R–R interval; SBP, systolic blood pressure; TG, triglycerides; VO_2max_, predicted maximal oxygen capacity; WC, waist circumference, *significant with *p* < 0.05.

Data presented as Spearman rank order correlation coefficient and *p*-value.

## Discussion

The main finding of this study was that a prescriptive exercise intervention supported by mHealth technology improved HRV by increasing and decreasing high and low frequency powers in normalized units, respectively. Additionally, the change in SBP was correlated with the change in R–R interval and HR.

Frequency domain analysis of HRV is commonly used since the different frequency bands are believed to reflect physiological processes (4). Specifically, it is generally accepted that the high frequency band reflects parasympathetic modulation, though there is some debate over the physiological significance of the low frequency band (4). The low frequency band may represent a combination of sympathetic and parasympathetic activity (4) or may be reflective of baroreflex activity (22, 23). There is evidence to suggest that when low frequency power is expressed in normalized units, it is reflective of sympathetic activity (24). Thus, the increase in high frequency power and decrease in low frequency power expressed in normalized units may indicate positive modifications in autonomic activity with an increase in parasympathetic and decrease in sympathetic activity following the intervention.

### Exercise and HRV

Exercise training is recommended as first-line treatment for MetS (2, 3) and has proved to effectively improve waist circumference, BP, and HDL in MetS populations (25). Additionally, lifestyle interventions combining diet and exercise reduced incident type 2 diabetes mellitus in a population of adults with MetS risk factors (26, 27). In the DaTA pilot study, the 8-week mHealth supported exercise intervention reduced DBP and total cholesterol and increased predicted VO_2max_ (16). There is some evidence to suggest that exercise may also have positive effects on HRV, but results are mixed. In an early study, young adults with mild hypertension completed 22 min of calisthenics followed by 20 min of jogging a minimum of five times per week (28). Upon completion of the intervention, resting R–R interval was increased and, similar to the present study, high and low frequency powers expressed in normalized units were increased and reduced, respectively, demonstrating improved autonomic balance (28). Exercise training interventions, of moderate-to-vigorous intensity in middle-aged to older men (13, 29, 30) and a lower intensity exercise intervention at 50% of VO_2max_ in post-menopausal women also showed improvements in HRV (31). One study in patients with type 2 diabetes mellitus with and without cardiac autonomic neuropathy showed improvement in HRV parameters following 6 months of aerobic exercise three times per week at 70–85% of HR reserve (32). Conversely, one study showed no change in HRV parameters at rest or during exercise in older men and women after 8 weeks of aerobic exercise training three times per week for 60 min each session (33).

Other studies found that while resting HRV was not altered with endurance training, HRV was altered during or after exposure to stressors (34, 35). In obese women with and without type 2 diabetes mellitus, 16 weeks of moderate intensity endurance training (65% VO_2max_) 4 days per week increased post-exercise HR recovery and post-exercise high and low frequency powers with no changes in resting HRV (34). Six months of exercise training 2 days per week for 70 min at moderate intensity did not affect resting HRV in patients with type 2 diabetes mellitus, but high frequency power in normalized units was increased and the ratio of low-to-high frequency powers was reduced during an orthostatic challenge (35). Thus, inclusion of an acute stressor to the study protocol may have revealed additional alterations in autonomic activity following the intervention.

Frequency of exercise training may be an important factor, as those with sessions five to six times per week (13, 28, 30) showed improved HRV, while in those with training sessions four times per week or less, results were mixed. In four studies that did not show changes in resting autonomic function, exercise training frequency was only two or three times per week or only one session per week was supervised and the remaining were completed at home, so compliance to the exercise protocol may not be as high as reported. Since the exercise prescription in the present study included attainment of 10,000 steps daily along with other more specific aerobic exercises at moderate-to-vigorous intensity, the frequent exercise bouts may have been responsible for the improvements in high and low frequency powers in normalized units. Unfortunately, only pedometer steps were recorded and other activities were not logged, so frequency and intensity of exercise completed throughout the intervention cannot be quantified.

Age may play an important role in the response of HRV to exercise. Lee and colleagues (36) used low dose atropine (which augments parasympathetic activity, as opposed to higher doses, which are inhibitory) to provoke autonomic changes in younger and older fit and unfit individuals. Resting R–R interval was increased more in fit compared to unfit individuals in the younger age group. However, the responses to atropine in the older age group were not affected by fitness status (36). Thus the lack of change in HRV in response to exercise training in older adults may be due to reduced function or sensitivity of the sinus node with age, which may not be modified with intervention. One study in older women showed that exercise improved HRV when 50, 100, or 150% of national exercise guidelines was completed. However, in women aged >60 year, there were no improvements at lower exercise doses, suggesting that a higher dose of exercise may be necessary to evoke autonomic changes in older adults (37). Though our sample was on average older, it included a range of ages. Interestingly, age was not correlated with change in any HRV parameter in this study.

In the Diabetes Prevention Program, an intensive lifestyle intervention including physical activity and a low fat diet reduced HR and increased HRV parameters indicative of overall or beat-by-beat variability (reflecting parasympathetic activity) compared to metformin or placebo (38). Importantly, reductions in HR and increases in HRV over time were associated with lower risk of incident diabetes, independent of weight loss, and physical activity in the lifestyle modification group, supporting the use of HRV or perhaps the change in HRV in response to an intervention, as a risk indicator (38). HRV was measured from 10 s ECG segments, so frequency domain parameters could not be examined, which also have important prognostic power. Nevertheless, these results are promising and suggest that lifestyle modifications are important for risk reduction in a diabetes prevention strategy. Improvements in autonomic function may have positive effects in the long-term. Longitudinal follow-up studies are warranted to examine the effects of improved HRV on prognosis.

### HRV and MetS

To our knowledge, this study was the first to examine correlation between the change in HRV parameters and the change in MetS risk factors over an intervention period. The change in SBP was strongly and inversely associated with changes in R–R interval and positively associated with changes in HR. These findings are in accordance with previous studies that have shown reduced BP and HR with endurance exercise training (39). The correlations between changes MetS risk factors and changes in HRV parameters over an intervention period are not the same as cross-sectional associations between 24 h HRV and MetS risk factors. Assoumou et al. (11) showed associations between hypertension and total power, but did not examine relationships with SBP and DBP individually. Their study showed a negative association between fasting plasma glucose and high frequency power of HRV (11), while the present study showed no associations between fasting plasma glucose and HRV parameters. When sex differences were examined triglycerides were only associated with HRV in men, but not women and fasting plasma glucose was negatively associated with high frequency power and positively associated with ratio of low-to-high frequency powers in women with no associations in men (11). In a cohort of men with MetS, all MetS risk factors except HDL were associated with HRV parameters (12). Notably, these authors showed that each one unit increment in the number of MetS components reduced low frequency power of HRV by 15% (12), highlighting the importance of treating and controlling all MetS components. Liao and colleagues (40) examined 2-min HRV in people with what they termed multiple MetS, which could be any combination of hypertension, type 2 diabetes mellitus, and dyslipidemia. Interestingly, they showed that the combination of risk factors presenting together determined the HRV outcome. When hypertension was present in combination with either type 2 diabetes mellitus or dyslipidemia, the reductions in HRV were additive, but when type 2 diabetes mellitus and dyslipidemia presented together, reductions in HRV were multiplicative (40). These findings may account for differences between the present study and others, as specific combinations of risk factors may be more important contributors to HRV than individual MetS components. The sample size for this pilot project was too small to include an analysis based on the change in MetS risk factors, but such analyses are important for future studies. Additionally, the present study was the first to examine associations between longitudinal changes in HRV and changes in MetS, which may be different than cross-sectional associations.

In this study, changes in waist circumference were not significantly correlated with changes in HRV. There was however, a strong trend (*p* = 0.06) for a negative association between the change in waist circumference and the change in high frequency power of HRV, suggesting that reductions in waist circumference may not be associated with improvements in HRV, but rather with declines in HRV. On the contrary, Hautala and colleagues (13) showed that an 8-week intervention with supervised exercise training sessions six times per week at 70–80% of maximum HR improved HRV. The change in body mass index was moderately correlated with the change in high frequency power following the subsequent 10-week home-based maintenance program, despite the fact that overall improvements in HRV were not maintained during this period (13). Furthermore, in a small sample of abdominally obese men and women, weight loss with a very low calorie diet increased total and low frequency power of HRV (41). However, despite weight maintenance over a 1-year period, improvements in HRV were not sustained (41). These studies suggest that improvements in HRV with lifestyle interventions may be transient. Long-term studies to determine effective strategies for maintenance of HRV improvements with exercise or weight loss interventions are needed.

### Limitations

This pilot study was conducted in a sample of highly motivated volunteers and may therefore lack external validation. Despite the fact that only 12 participants had acceptable HR data for 24 h HRV analysis, modifications were seen in high and low frequency powers of HRV when expressed in normalized units. However, this analysis was underpowered and a larger sample size would have been ideal and may have shown greater change. Although Suunto activity monitor has been validated, our participants had difficulties putting the monitor on properly to collect heart period. Additionally, some participants reported discomfort and removed the monitor during the nighttime period due to disturbed sleep. Traditional Holter monitors may be preferable to improve data quality and participant comfort. This exploratory pilot study used correlation analysis to examine relationships between MetS risk factors and HRV parameters. Unfortunately, correlation cannot show direction of association. Additionally, previous studies have shown sex differences in associations between MetS risk factors and HRV (11), but the sample for this pilot study was not sufficiently powered to examine sex differences. Nevertheless, potentially important relationships were identified in this sample. Future studies examining relationships and sex differences in a larger population are warranted.

In conclusion, an 8-week exercise prescription supported by mHealth technologies for biometric and activity monitoring improved 24 h HRV profile with increased high frequency power of HRV in normalized units and reduced low frequency power of HRV in normalized units, suggesting improved autonomic balance. Additionally, changes in SBP were associated with changes in R–R interval and HR.

## Conflict of Interest Statement

The authors declare that the research was conducted in the absence of any commercial or financial relationships that could be construed as a potential conflict of interest.
